# Structural characteristics and regulatory mechanisms of the ocular surface microbiota and their relationship with ocular surface health

**DOI:** 10.3389/fcimb.2026.1938863

**Published:** 2026-10-21

**Authors:** Tianyao Zhao, Yingjuan Sun, Xiaodan Jiang, Yue Feng, Jing Ding, Xuemin Li, Aihua Deng

**Affiliations:** 1 Department of Ophthalmology, Peking University Third Hospital, Beijing Key Laboratory of Restoration of Damaged Ocular Nerve, Beijing, China; 2 Fangshan Hospital, Beijing University of Chinese Medicine, Beijing, China; 3 State Key Laboratory of Animal Nutrition and Feeding, Ministry of Agriculture and Rural Affairs Feed Industry Centre, China Agricultural University, Beijing, China

**Keywords:** dry eye, immune homeostasis, meibomian gland dysfunction, multi-omics, ocular surface microbiota

## Abstract

This review delineates the spatial distribution of ocular surface microbiota (OSM) and its dynamic interplay with ocular health. Culture-independent surveys employing 16S rRNA and metagenomic sequencing reveal an ultra-low-biomass ecosystem characterized by restricted α-diversity. *Firmicutes*, *Actinobacteria* and *Proteobacteria* are the dominant phyla. *Corynebacterium*, coagulase-negative staphylococci, *Propionibacterium* and *Streptococcus* show high prevalence and temporal stability. These four genera form a conserved core. Their relative abundance is continuously reshaped by geography, lifestyle, age, sex, delivery mode, contact lens wear and systemic diseases such as dry eye and diabetes. Emerging evidence implicates host-microbe crosstalk, encompassing Toll-like receptor (TLR) and NOD-like receptor (NLR) mediated sensing of pathogen-associated molecular patterns (PAMPs), secretory immunoglobulin A (SIgA) neutralization, and a putative gut-eye immunoregulatory axis, in ocular immune maturation and the maintenance of immune homeostasis, although direct causal relationships remain to be established. Under physiological conditions, competitive exclusion and negative feedback maintain microbial equilibrium. However, surgical trauma, antibiotics or corticosteroids have been associated with destabilization of this niche, under which commensals may acquire virulence genes, form biofilms and transition into opportunistic pathogens implicated in infectious keratitis, exacerbation of meibomian gland dysfunction and increased dry eye severity. Consequently, high-resolution profiling of OSM composition and function may help clarify disease mechanisms and holds promise for furnishing microbial biomarkers for precision diagnosis and personalized therapeutic intervention.

## 1 Introduction

The human body constitutes a complex ecosystem in which microbial communities reside in the gastrointestinal tract, oral cavity, nasal mucosa, and urogenital tract. Perturbations or transient expansions of these microbiotas have been associated with a spectrum of diseases (Lloyd-Price et al., 2016). The ocular surface denotes the entire mucosal epithelium covering the globe, delimited by the gray line of the upper and lower eyelids. It encompasses the corneal epithelium, limbal epithelium, conjunctival epithelium, and the overlying tear film; by extension, it also includes the eyelid skin, lacrimal apparatus, and nasolacrimal drainage system. Because the ocular surface communicates directly with the external environment, it is colonized by a diverse array of microbial communities (McDermott, 2013). The commensal, non-pathogenic microorganisms that reside on the conjunctival and corneal surfaces are collectively designated the ocular surface microbiota (OSM). This consortium typically comprises bacteria, fungi, and other microbial taxa whose homeostatic interplay is intimately linked to ocular surface health. In healthy individuals the OSM constitutes a complex and highly dynamic ecosystem whose composition is shaped by a multitude of host-related and environmental factors. Compared with the microbiotas that colonize other mucosal niches of the human body, the OSM is distinguished by its uniquely restricted diversity and biomass. Consequently, its influence on host physiology and immunity may differ in both magnitude and mechanism from those exerted by microbiotas at alternative mucosal sites (Chen et al., 2021; Peng et al., 2022). The presence of commensal microorganisms on the ocular surface and their modulatory effects on the local immune apparatus constitute a research question that remains insufficiently resolved. It should be emphasized that much of the available evidence derives from animal models, small-cohort microbiome profiling studies, or indirect systemic correlations. Accordingly, the reported relationships between the OSM, ocular surface immunity and disease are predominantly associative rather than causative. Against this background, the primary aim of this review is to synthesize current evidence on the structural characteristics of the OSM, including its composition, diversity and temporal stability, to critically evaluate the host-related, environmental and methodological factors associated with its variation, and to summarize the proposed mechanisms linking the OSM to ocular surface immune regulation and disease. We further discuss the major limitations of current ocular surface microbiome research and outline priorities for future studies, with the ultimate goal of providing a framework for interpreting OSM data and for exploring microbiota-informed strategies to preserve ocular surface health.

## 2 Composition and diversity of the ocular surface microbiota in healthy populations

The composition and diversity of the ocular surface microbiota represent a central axis of investigation for ocular surface health. Among its constituents, bacteria constitute the dominant fraction. Although fungi and viruses are recovered far less frequently and in lower taxonomic variety, they are increasingly thought to contribute to the dynamic equilibrium that characterizes the microbiota. In a large survey conducted by Tao et al., 8,224 conjunctival sac samples from central China were submitted to culture-based identification. *Staphylococcus epidermidis* was recovered from 39.7% of specimens, followed by *Streptococcus pneumoniae* (4.5%) and *Staphylococcus aureus* (2.7%), establishing these species as the most prevalent cultivable bacteria in that regional population (Tao et al., 2017). Doan et al. employed 16S rDNA sequencing coupled with quantitative PCR to characterize the bacterial consortia residing in the conjunctival sacs of healthy volunteers, with the explicit aim of delineating the compositional profile of the ocular surface microbiota (Doan et al., 2016). Specimens were collected from the superior and inferior conjunctivae, periocular skin, and oral mucosa. Metagenomic datasets were then integrated to provide a high-resolution taxonomic description of the bacterial communities at each sampled anatomical site. Metagenomic datasets were further interrogated to determine the presence of fungal and viral reads within the BRiSK sequence library. The analyses identified *Corynebacterium*, *Propionibacterium*, coagulase-negative staphylococci (CNS), and *Streptococcus* as the putative core taxa that consistently constitute the conjunctival sac microbiota of healthy individuals. Quantitative comparison revealed a higher bacterial DNA load in the inferior conjunctiva than in the superior conjunctiva. Among the viral sequences recovered, Torque teno virus (TTV), a non-enveloped, circular single-stranded DNA virus, was detected. The same study further demonstrated that TTV is highly prevalent within the ocular surface microbiota of healthy individuals: sixty-five percent of the eighty conjunctival samples harbored at least one TTV genome copy per one hundred epithelial cells. This finding indicates that TTV may exert a biological influence on ocular health and disease.

Huang et al. (2016) employed high-throughput 16S rDNA sequencing to profile the OSM of healthy volunteers. Their analysis delineated a set of genera that appear to constitute the core microbiota of the healthy human eye, with *Corynebacterium* (28.22%), *Pseudomonas* (26.75%), *Staphylococcus* (5.28%), *Acinetobacter* (4.74%), and *Streptococcus* (2.85%) showing the highest relative abundances. Although several genera were shared across subjects, the relative abundance of each taxon differed markedly among individuals. The investigation did not, however, provide absolute quantitative data, nor did it account for environmental exposure, occupation, or age, factors that are needed to fully interpret compositional variation and the contribution of the OSM to eye health. Evidence indicates that the ocular surface microbiota of healthy individuals is characterized by low alpha diversity. At the individual level, certain bacterial species remain detectable across multiple sampling time points, suggesting the presence of a subject-specific core microbiome. Nevertheless, both the taxonomic profile and the relative proportions of these organisms vary considerably among persons and fluctuate over time, implying that the composition of the OSM is shaped by host-specific factors. Within each conjunctival sac specimen, the genera most frequently detected by molecular methods also yielded the highest recoverable counts on culture plates, with individual eye samples ranging from 2 to 170 colony-forming units (CFU) (Ozkan et al., 2017). Wang et al. (2020a) profiled the OSM of 45 healthy volunteers (90 eyes) using both culture-based methods and high-throughput sequencing. They identified two phyla, *Basidiomycota* and *Ascomycota*, along with five genera: *Malassezia, Rhodotorula, Davidiella, Aspergillus*, and *Alternaria*. These taxa made up more than 80% of the total fungal sequences. They were present in over 80% of conjunctival sac samples. Together they form a proposed core fungal community on the healthy ocular surface. At present, data on the normal ocular surface mycobiota remain scarce. This study therefore establishes a baseline for future investigations into the potential roles of fungal communities in maintaining ocular health and in precipitating disease.

In summary, the OSM of healthy individuals displays shared features, most notably the repeated detection of *Staphylococcus* spp., suggesting that this genus may perform functions critical to ocular surface homeostasis. Even so, the precise profiles reported by different studies vary considerably. These variations are likely linked not only to differences in environmental exposure, lifestyle, and underlying health status, but also to methodological heterogeneity, including differences in sampling sites and techniques, DNA extraction protocols, the 16S rRNA gene variable regions targeted, sequencing platforms and bioinformatic pipelines, as well as the contamination risk inherent to low-biomass samples. The principal studies characterizing the OSM in healthy and diseased populations, together with their cohorts, methods and main findings, are summarized in **Table 1**.

**Table 1:** Summary of principal studies on the OSM.

Study (year)	Population	Sample size		Method	Principal microbial/taxa identified/main findings
Eder et al., 2005	Neonates, Argentina and Paraguay	Two hospital centers		Culture	CNS most frequent; higher culture-positive rate after vaginal than caesarean delivery
Doan et al., 2016	Healthy volunteers, USA	Conjunctiva, periocular skin and oral mucosa samples		16S rRNA sequencing qPCR, metagenomics	*Corynebacterium, Propionibacterium*, CNS and *Streptococcus* as core taxa; resident DNA virome including TTV
Huang et al., 2016	Healthy volunteers, China	31 conjunctival swab samples		16S rRNA (V3-V4) Illumina sequencing	*Corynebacterium* (28.22%), *Pseudomonas* (26.75%), *Staphylococcus* (5.28%), *Acinetobacter* (4.74%), *Streptococcus* (2.85%)
Tao et al., 2017	Healthy individuals, central China	8,224 conjunctival sac samples		Culture-based identification	*Staphylococcus epidermidis* (39.7%), *Streptococcus pneumoniae* (4.5%), *Staphylococcus aureus* (2.7%)
Ozkan et al., 2017	Healthy adults, Australia	Longitudinal sampling at multiple time points		16S rRNA sequencing and culture	Subject-specific core with temporal stability; 2-170 CFU per eye
Wen et al., 2017	Healthy adults, China	Adult cohort		Shotgun metagenomic sequencing	Sex-related differences: *Propionibacterium acnes* and *Staphylococcus epidermidis* higher in men; *Escherichia coli* more frequent in women
Wang et al., 2020a	Healthy volunteers, China	45 volunteers (90 eyes)		Culture and high-throughput sequencing (fungi)	*Basidiomycota* and *Ascomycota*; *Malassezia, Rhodotorula, Davidiella, Aspergillus, Alternaria* (>80% of fungal sequences)
Deng et al., 2020	Healthy young Han Chinese, three cities		172 volunteers (Guangzhou, Wenzhou, Beijing)	Deep shotgun metagenomic sequencing	City-specific profiles; *Propionibacterium* dominant in Guangzhou, *Pseudomonas* in Beijing; city-specific metabolic pathways
Ke et al., 2020	Healthy children, China		6,440 children	Culture	*Staphylococcus* (37.4%), *Corynebacterium* (30%), *Streptococcus pneumoniae* (21.4%); Gram-positive dominance
Jiang et al., 2023	Healthy adolescents East China		1,258 adolescents (2,516 eyes)	Culture	*Staphylococcus epidermidis, Streptococcus* and *Staphylococcus aureus* most frequent; *Staphylococcus epidermidis* detection rate correlated with age
Jiang et al., 2018	MGD patients and controls, China		70 patients (140 eyes)	16S rRNA sequencing and culture	*Staphylococcus epidermidis* most common; *Corynebacterium macginleyi* enriched in severe MGD (26.3%)
Zou et al., 2023	Dry eye patients of varying severity, China		199 patients (aged 4-84 years)	16S rRNA sequencing	OSM diversity decreased and composition homogenized with increasing dry eye severity
Delbeke et al., 2023	Ocular surface samples, Belgium		Method-comparison cohort	Comparison of DNA extraction protocols	Microbial composition significantly affected by extraction protocol, even after decontamination
De Luca et al., 2026	Contact lens wearers		Narrative review	Literature review	Contact lens use associated with alterations of the ocular surface bacterial microbiome

## 3 Factors influencing the composition of the ocular surface microbiota

### 3.1 Environmental and lifestyle factors

Environmental factors such as geographic location and prevailing climate markedly influence the diversity and abundance of the OSM. Deng et al. recruited 172 healthy volunteers from three Chinese cities: Guangzhou, Wenzhou and Beijing. Conjunctival sac swabs were collected from each participant and analyzed by deep metagenomic shotgun sequencing (Deng et al., 2020). The analysis revealed distinct conjunctival sac microbial profiles among the three cities. Beijing samples exhibited higher alpha diversity and a unique community structure that separated them from the Guangzhou and Wenzhou cohorts. At the genus level, the dominant taxa also differed: *Propionibacterium* was most abundant in Guangzhou samples, whereas *Pseudomonas* predominated in those from Beijing. Functional prediction further showed city-specific metabolic pathways, implying that local environmental variables shape both the taxonomy and the functional capacity of the OSM. Short-term travel to cities within the same province did not measurably alter the composition or predicted metabolic profile of the OSM. In contrast, prolonged residence in a different province was associated with detectable shifts in community structure. Dietary pattern also emerged as a relevant variable. Individuals with markedly different eating habits harbored distinct OSM profiles, implying that nutritional environment contributes to microbiota assembly at the ocular surface. Zhang et al. (2012) used cluster sampling to compare the conjunctival sac bacterial community between Qiang and Han patients with dry eye living in Sichuan, China. *Corynebacterium* and *Staphylococcus epidermidis* were present in both ethnic groups. In contrast, the isolation rate of *Skermanella* was markedly higher in Qiang participants, whereas this genus was absent or rarely detected in Han participants. These differences suggest that the OSM is shaped by ethnicity-related variations in living environment, cultural habits, and genetic background.

### 3.2 Age

Age has been consistently associated with differences in OSM composition, and both diversity and abundance appear to shift across the human lifespan. Colonization of the conjunctival sac is thought to begin at birth. Eder et al. (2005) showed that CNS are the organisms most frequently recovered from the conjunctival sac of neonates. The rate of positive bacterial cultures was significantly higher in infants delivered vaginally than in those born by caesarean section. Among neonates delivered vaginally, the most commonly isolated bacteria from the conjunctival sac were CNS, *Propionibacterium* spp., and *Corynebacterium* spp. In contrast, the most frequently isolated bacteria from neonates delivered by caesarean section were CNS, *Propionibacterium* spp., and *Staphylococcus aureus*. The establishment of the OSM in neonates may be essential for appropriate immune development of the ocular surface. Subsequently, factors such as geographic location, environment, and dietary habits gradually influence the composition of the conjunctival sac microbiota. Ke et al. examined the OSM of 6440 children, with a positive rate of bacterial culture at 21.8%. The study revealed that the OSM of children is primarily composed of Gram-positive bacteria, with the most common genera being *Staphylococcus* (37.4%), *Corynebacterium* (30%), and *Streptococcus pneumoniae* (21.4%) (Ke et al., 2020). Jiang et al. (2023) analyzed the conjunctival sac microbiota of 1258 adolescents under 18 years old (2516 eyes) in China and similarly found that the microbiota was dominated by Gram-positive cocci. The three most frequently detected genera were *Staphylococcus epidermidis*, *Streptococcus*, and *Staphylococcus aureus*. The detection rate of *Staphylococcus epidermidis* was positively correlated with age. In children aged 0–6 years, the proportion of *Streptococcus* was higher than that of *Staphylococcus aureus*. Therefore, the bacterial community structure varies with age, a process that may be influenced by factors such as living environment and lifestyle. The composition of OSM in the elderly differs from that in the young. Katzka et al. found that patients over 65 years of age had higher alpha diversity in OSM (Katzka et al., 2021). Similar to this study, the studies of Zhou et al. and Tao et al. also observed age-related changes in OSM, with changes in the positive rate of bacterial culture and the diversity of bacterial flora from children to adults (Zhou et al., 2014; Tao et al., 2017)). This change may be related to age-related changes in immune function. With the increase of age, the function of the immune system gradually weakens, which may lead to the corresponding changes in the composition of OSM in the elderly. Terzulli et al. verified this hypothesis through animal experiments and found that the composition and number of bacteria colonizing conjunctival sacs changed with the age of mice (Terzulli et al., 2015). Age-related alteration of the microbiome has been proposed as one of the hallmarks of aging (Bradley and Haran, 2024). Whether the OSM of elderly individuals exhibits characteristic features, how such features relate to aging-associated physiological processes, and whether they are linked to age-related ocular diseases remain open questions that warrant further investigation.

### 3.3 Gender

Sex-related differences in OSM composition have been reported, although the findings are not fully consistent across studies. Kawata et al. found that the culture-positive rate of OSM in elderly women may be higher than that in elderly men (Kawata and Matsuo, 2017). The results of Xu et al. were similar in that the positive rate of bacterial culture of conjunctival sac in women was higher than that in men in ophthalmic perioperative patients (Xu and Zhang, 2022). However, studies by Tao et al. and Kuo et al. found a higher rate of bacterial isolation in men than in women, which is consistent with a higher risk of endophthalmitis in male patients (Tao et al., 2017; Kuo et al., 2020). A comparison of the methodologies used suggests that ethnic differences of the study populations, together with different inclusion and exclusion criteria and different culture and identification methods, may account for these discrepant results. Ozkan et al. found that there was no significant difference in bacterial species and quantity between male and female OSM in healthy populations, but the Shannon diversity index of male OSM was significantly higher than that of female OSM, indicating that the composition of male OSM was more complex and abundant than that of female OSM, suggesting that the OSM of males may be more diverse than that of females (Ozkan et al., 2017). Through metagenomic sequencing, Wen et al. found that there were significant differences between males and females in the *β-diversity* of OSM (Wen et al., 2017). The relative abundance of *Propionibacterium acnes* and *Staphylococcus epidermidis* in the conjunctival sac was significantly higher in men than in women, while *Escherichia coli* was more common in the conjunctival sac in women. Relatively few studies have focused on sex-related differences of the OSM, and comparing such differences may help to delineate the complexity of the OSM more comprehensively. Future studies could investigate the underlying biological mechanisms, for example whether sex hormones such as androgens and estrogens influence the composition and function of the OSM through modulation of the immune system and the ocular surface microenvironment.

### 3.4 Physical health condition

The composition of the OSM may change under different health conditions. For instance, ocular surgery, contact lens wear, inflammation, other ocular diseases, and systemic diseases have all been associated with significant alterations in the OSM. Hu et al. (2022) found that through perioperative management prior to intravitreal injection, the composition and relative abundance of the OSM underwent obvious changes. For example, *Proteus* spp. were detected in the conjunctival sac of patients who received multiple rounds of perioperative management. Wearing contact lenses, especially prolonged wear, may lead to changes in the species and quantities of certain bacteria within the OSM. A study by Willcox et al. (2011) specifically pointed out that after contact lens wear, the bacterial carriage status on the contact lens surface and ocular surface is associated with the occurrence of adverse reactions. Specifically, individuals carrying Gram-positive bacteria (such as CNS or *Corynebacterium* spp.) are more likely to develop contact-lens-induced peripheral ulcers and asymptomatic infiltrates. Meanwhile, the carriage of Gram-negative bacteria (e.g., *Haemophilus influenzae*) is associated with contact-lens- induced acute red eye symptoms. Therefore, during the wear and care of contact lenses, the natural balance of the OSM may be disrupted, which may promote the overproliferation of opportunistic pathogens and has been linked to an increased risk of ocular infections. A recent literature review similarly concluded that contact lens use is associated with measurable alterations of the OSM, while emphasizing that the clinical significance of these shifts and their causal role in contact-lens-related complications remain to be determined (De Luca et al., 2026). As for whether contact lens wear affects the response of corneal epithelial cells to microorganisms, and whether it promotes bacterial adhesion and infection, these questions require further research to clarify.

The severity of dry eye disease is significantly associated with the diversity and composition of the OSM. Our previous study (Jiang et al., 2018) used 16S rRNA gene sequencing technology to analyze 70 patients (140 eyes) with MGD of varying degrees, aged 17–60 years. First, the study found that *Staphylococcus epidermidis* was the most commonly isolated bacterium, particularly in MG secretions. Compared with the conjunctival sac, the bacterial isolation rate in MG secretions was significantly higher. Furthermore, the bacterial isolation rate in MG secretions of patients with MGD was significantly higher than that in the control group. In the control group and the mild MGD group, the bacterial composition was relatively simple, mainly consisting of Gram-positive bacteria, including the genera *Staphylococcus*, *Corynebacterium*, and *Micrococcus*. With the increase in the severity of MGD, the bacterial species became more complex. More bacterial species were isolated from the moderate and severe MGD groups. Among them, *Corynebacterium macginleyi* was more prominently detected in the severe MGD group, with an isolation rate as high as 26.3%. These results indicate that dry eye patients, especially those with MGD, exhibit significant differences in the bacterial distribution of the conjunctival sac and MG compared with normal individuals, and these differences may be associated with the pathophysiological mechanism of MGD. Based on these findings, we propose a novel bacterial severity grading system for MGD, which provides new insights for clinical treatment. However, the research results by Zou et al. exhibit certain differences from ours (Zou et al., 2023). They analyzed the OSM of 199 dry eye patients with varying severity using 16S rRNA gene sequencing technology, and the age range of the study participants was 4–84 years. This study found that as the severity of dry eye disease increased, the diversity of the OSM decreased, and the species composition tended to be more homogenized. Although the two studies showed differences in the analysis of the microbial composition of patients with dry eye disease of varying severity, these differences may be related to the age of the participants and environmental factors. Nevertheless, both sets of results support an association between the OSM and the occurrence and progression of dry eye disease. Studies have shown that certain tear proteins in the tears of patients with dry eye disease, such as lactoferrin, lysozyme, polymeric immunoglobulin receptor, and lactoperoxidase, are selectively downregulated. These proteins have bactericidal activity, and their downregulation further supports the rationality of the association between the OSM and the ocular surface barrier (Karnati et al., 2013). Patients with diabetes have an increased risk of developing dry eye disease. The hyperglycemic environment provides sufficient nutrients for ocular surface microorganisms, which may lead to increased diversity of the OSM in diabetic patients. Among these microorganisms, certain bacteria may promote the expression of inflammatory factors by producing toxins and superantigens, thereby exacerbating ocular surface inflammation (An and Zou, 2023; Zhang et al., 2021). In addition, other diseases and conditions affecting the ocular surface may also induce changes in the OSM. These include blepharitis, Sjögren’s syndrome, allergic conjunctivitis, graft-versus-host disease, keratoconus, Stevens-Johnson syndrome, thyroid-associated ophthalmopathy, and changes in the gut microbiome (Kim et al., 2022; Zarzuela et al., 2022; Bohlen et al., 2024; Rocha-de-Lossada et al., 2023). By analyzing the composition and diversity changes of the OSM, it may be possible to provide new biomarkers for the diagnosis of eye diseases and help develop targeted treatment strategies. Meanwhile, changes in the OSM are not only associated with eye diseases but may also be related to systemic diseases, suggesting a complex interaction between the OSM and overall health. Further research could reveal the mechanisms by which systemic diseases affect the OSM and develop new regulatory strategies.

### 3.5 Collection and detection methods

The first study on the OSM dates back to the 1970s. At that time, culture-based techniques were primarily used for the identification of microbial colonies. Next generation sequencing (NGS) technologies have advanced significantly in recent years. Key methods in this field include 16S rRNA sequencing, shotgun metagenomics, and RNA-based deep metagenomic sequencing. Research conducted over the past decade has revealed that these NGS approaches are better able to characterize the microbial environment of the ocular surface. However, factors such as different sampling methods, sampling regions, sampling depths, as well as microbial detection and identification techniques, all exert significant impacts on the research results regarding the compositional analysis of the OSM (Wang et al., 2020b). In culture-based studies, the OSM is primarily composed of Gram-positive bacteria, including the genera *Staphylococcus*, *Streptococcus*, *Corynebacterium*, and *Propionibacterium*. In contrast, the abundance of Gram-negative bacteria is relatively low; these bacteria are mainly from the genera *Haemophilus*, *Moraxella*, and *Klebsiella* (Eguchi et al., 2021). While culture-based studies may underestimate the diversity of the OSM, the same methods can yield a greater variety and quantity of microorganisms when applied to sampling and culturing other regions. These regions include the skin, oral mucosa, and intestinal tract. This finding indicates that the composition of the OSM is less diverse compared to these aforementioned areas. Huang et al. (2016) employed Illumina high-throughput sequencing technology to sequence all bacterial 16S rDNA V3-V4 hypervariable regions in conjunctival swab samples. A total of 840,373 high-quality sequences were obtained from 31 samples. These sequences revealed substantial microbial diversity, which was classified into 25 phyla and 526 genera. At the genus level, *Corynebacterium*, *Pseudomonas*, *Acinetobacter*, *Streptococcus*, and *Micrococcus* exhibited relatively high detection rates. These genera may represent the “core genera” of the normal OSM. The study by Zysset-Burri et al. employed metagenomic sequencing techniques to investigate the conjunctival sac samples from 20 elderly individuals (Zysset-Burri et al., 2021). This inquiry yielded approximately 96.4 million non-human high-quality sequences, with an average of about 2.4 million sequences per sample. In the conjunctival sacs of these elderly subjects, the most prevalent bacterial phyla were *Actinobacteria* and *Proteobacteria*. Among the diverse genera identified, *Propionibacterium*, *Arthrobacter*, and *Corynebacterium* were the most abundant. Metagenomic sequencing, in addition to taxonomic analysis, enables functional annotation and analysis. It can identify microbial metabolic pathways and functional genes, offering a comprehensive view of microbial communities in samples. However, it is currently unclear whether the detected sequences represent live microbial communities in the conjunctival sac or dead microorganisms inhibited by competition or cleared by the immune system. Moreover, variations in OSM identification methods across different studies have led to difficulties in comparing and summarizing research findings. Several factors are likely to account for the divergent microbial compositions reported in the literature. First, the ocular surface is an ultra-low-biomass niche, in which contaminant DNA derived from sampling swabs, extraction kits and laboratory reagents can constitute a substantial fraction of the sequences obtained and may distort taxonomic profiles. Second, no standardized protocol exists: published studies differ in sampling site (superior versus inferior conjunctiva, eyelid margin or tear film), sampling device, DNA extraction and purification methods, the 16S rRNA gene variable regions targeted, sequencing platforms, and bioinformatic pipelines and reference databases, each of which introduces systematic bias (Katzka et al., 2021; Delbeke et al., 2023). Third, geographical and ethnic variability of the study cohorts further limits direct comparability (Deng et al., 2020). These methodological and biological sources of variation should therefore be considered when interpreting apparently conflicting compositional data.

Although various detection techniques have their unique advantages and limitations, researchers can select suitable OSM detection methods according to specific research objectives. Delbeke et al. compared the reproducibility of different DNA extraction protocols and their impact on research results and found that the differences in microbial composition caused by different extraction protocols are significant, even after decontamination (Delbeke et al., 2023). They suggested that appropriate DNA extraction protocols should be chosen based on specific research questions. Kuo et al. introduced a model combining dot hybridization assay with traditional cultivation and metagenomics (Kuo et al., 2020). This model assesses ocular surface microbial load, detects pathogens, and monitors the *mecA* antibiotic resistance gene. By tracking OSM changes, it offers personalized treatment to reduce postoperative infection risks. However, the model’s effectiveness awaits validation through larger, more diverse sample studies. In recent years, integrated multi-omics analysis has become a hot spot in life sciences research. Zysset-Burri et al. (2021) first explored the correlation between the classification and functional characteristics of OSM and the regulatory functional characteristics of the tear proteome in humans. The study revealed that *Firmicutes* showed a positive correlation with fatty acid binding function. *Arthrobacter* and vitamin B1 synthesis were positively correlated with the upregulation of antibacterial humoral reactions. The biosynthesis of heme, L-arginine, and L-citrulline was positively correlated with the upregulation of sensory perception of chemical stimuli. Integrated Multi-omics analysis provides significant microbial and functional information for biomarker research in ocular surface related diseases, aiding in the development of novel disease markers and targeted prevention and care strategies.

## 4 The interaction between the OSM and host immunity

The ocular surface, as an “immunologically privileged” area, not only effectively carries out immune defense but also strictly regulates to avoid excessive immune responses (Chen et al., 2022). This process may involve three key mechanisms. First, the intact ocular surface epithelial cells, such as the corneal epithelium, form a powerful barrier against microbial invasion. Mechanical defense mechanisms, through actions like blinking and tear secretion, can effectively clear potential pathogenic factors from the ocular surface. Second, tear fluid contains a range of antimicrobial components such as lysozyme, lactoferrin and defensins. These molecules inhibit and kill pathogens directly. Third, the ocular surface harbors immunocompetent cells. Neutrophils, lymphocytes and plasma cells that secrete secretory IgA all participate in pathogen defense through innate and adaptive responses. Together, these mechanisms form an integrated defensive network that maintains the homeostasis and health of the ocular surface microenvironment (Taube et al., 2015). Within this framework, the survival strategies of the OSM and their influence on ocular surface immunity deserve detailed investigation.

Given the markedly lower biomass and taxonomic richness of the ocular surface relative to other mucosal sites, the influence of the OSM on ocular health may share certain common features with microbiota at distant sites, yet it is more likely to exhibit distinct characteristics (Ozkan et al., 2017). Several investigators have proposed that the OSM may constitute a microbial barrier that, through continuous dialogue with the host immune system, triggers calibrated ocular surface immune responses and modulates inflammatory tone, thereby potentially contributing to tissue homeostasis (Aragona et al., 2021). Specific microbial constituents have been proposed to stimulate local antigen-presenting cells and to promote the development of adaptive immunity, processes that may reinforce host defense but also foster epithelial repair and regeneration. Concurrently, commensal species may restrain opportunistic pathogens via competitive exclusion, a mechanism that has been associated with a reduced risk of ocular infection (Li et al., 2020).

### 4.1 Activation of the ocular surface immune system by the OSM

Evidence derived mainly from animal models suggests that OSM-driven maturation of ocular surface immune cells contributes to protection against invading pathogens, although direct evidence in humans remains limited. The shielding mechanism directed at the surface polysaccharide PNAG of *Staphylococcus aureus* is executed through ICAM-1-dependent recruitment of polymorphonuclear leukocytes, the presence of fully competent CD4+ T cells, intact IL-17 receptor signaling and the production of IL-22. In germ-free mice the ocular surface fails to mobilize LY6G+ inflammatory cells and therefore cannot mount an effective defense against *Staphylococcus aureus* challenge. After three weeks of co-housing with conventionally raised animals, however, these formerly germ-free mice acquire a microbiota and regain full protective capacity against infection (Zaidi et al., 2014). This murine study suggests a potentially central role of the OSM in mediating adaptive immunity during *Staphylococcus aureus* keratitis, although its relevance to the human ocular surface awaits confirmation. T cells are central to autoimmune pathogenesis. The OSM is thought to modulate ocular immunity in part by influencing the activation of these cells. Microbial surface ligands bind Toll-like receptors (TLRs) on ocular surface tissue. This engagement initiates intracellular signaling cascades. The TLR family is evolutionarily conserved within the innate immune system. It also acts as a molecular bridge to adaptive immunity. TLRs recognize pathogen-associated molecular patterns (PAMPs). These patterns include bacterial flagellin, lipopolysaccharide and double-stranded RNA. Through this recognition, the receptors form the first line of host defense against microbial pathogens. TLRs are deployed either on the plasma membrane or in intracellular compartments. TLR1, TLR2, TLR5 and TLR6 reside at the cell surface, whereas TLR3, TLR7, TLR8 and TLR9 function within endosomes. TLR4, the principal lipopolysaccharide sensor, is unusual in that it operates both at the surface and in endosomal membranes. Its location dictates which ligand it engages and which signaling pathway it recruits (Chang and Winn, 2023). TLRs can discriminate between commensals and pathogens by recognizing discrete PAMPs derived from diverse microorganisms (Kawai et al., 2024). TLRs form a family of transmembrane sentinels that populate the ocular surface. The innate immune system uses these receptors to detect PAMP as well as lipoteichoic acids, lipoproteins and other bacterial products. When a TLR recognizes its cognate ligand, two receptor molecules dimerize on the plasma membrane. This pairing is the critical first step that launches intracellular signaling. After activation, the cytoplasmic tail of the TLR engages the adaptor myeloid differentiation primary response protein 88 (MyD88). This interaction nucleates a signaling platform that recruits and activates IRAK kinases, thereby initiating a phosphorylation cascade (Galletti et al., 2017). The cascade culminates in the activation of NF-κB and mitogen-activated protein kinase (MAPK) signaling modules. These pathways switch on a coordinated transcriptional program that drives expression of inflammation-related genes. Consequently, epithelial and resident immune cells synthesize and secrete pro-inflammatory cytokines such as tumor necrosis factor-α (TNF-α), IL-6 and IL-1β, together with chemokines and growth factors. After synthesis on the rough endoplasmic reticulum, these mediators are processed in the Golgi apparatus, packaged into vesicles and released to the extracellular space. The secreted molecules recruit and activate dendritic cells, helper T lymphocytes and B cells, thereby orchestrating local immune responses (Miller and Iovieno, 2009). Experimental evidence demonstrates that ocular staphylococci can stimulate epithelial immune cells to modulate both the composition and the production of mucins, while simultaneously enhancing interactions with pattern-recognition receptors and Toll-like receptors to reinforce mucosal immunity (Kumar et al., 2006). Nucleotide-binding oligomerization domain-like receptors (NLRs) constitute a key intracellular sensor family of the innate immune system. They detect cytosolic pathogens and danger signals. Oh et al. reported that mRNA levels of NOD2 and NLRP3 are markedly elevated in actively infected human corneas, indicating that NLRs may play an important role in corneal innate immunity (Oh et al., 2017). Jiang et al. (2024) showed that NLRP12 overexpression significantly reduces viral replication in a murine model. The underlying mechanism involves inflammasome-mediated pyroptosis and downstream IL-18 and IFN-γ signaling. Collectively, these studies suggest that NLRs recognize pathogens and modulate immune responses during infectious keratitis. At present, however, no data are available on NLR expression in healthy human corneas or on how the OSM influences their activation state.

IgA plays a pivotal role in host defense. At the ocular surface, secretory immunoglobulin A (SIgA) is the principal antibody. It is a dimeric IgA produced by plasma cells located beneath the lacrimal gland epithelium. The molecule neutralizes pathogens and blocks their adhesion to ocular surface epithelial cells (Kugadas et al., 2017). Two subclasses exist. IgA1 predominates in serum, whereas IgA2 is the major subclass found in mucosal secretions such as such as saliva, tears and intestinal fluid (De Sousa-Pereira and Woof, 2019). This distribution profile equips IgA2 with a central role in mucosal immunity, particularly within the ocular surface immune response. Allansmith et al. transferred germ-free rats to a conventional environment and documented a marked rise in IgA and IgM producing B cells in the lacrimal gland (Allansmith et al., 1987). Within four weeks the cell numbers reached levels indistinguishable from those of normal rats. Although the study did not directly examine the influence of the OSM on secretory IgA output, it established that environmental cues are associated with lacrimal B cell activation, indirectly implicating the OSM as a potential facilitator of this process. Lan et al. demonstrated that commensals colonizing the human conjunctiva stimulate a selective increase of tear SIgA (Lan et al., 1997). The antibody cross reacts with *Staphylococcus aureus* cell wall antigens but not with Gram-negative bacteria, indicating that SIgA restricts the load of Gram-positive commensals while simultaneously neutralizing pathogens such as *Staphylococcus aureus*, thereby preserving ocular surface health. In addition, tear mucins and surfactant protein D bind microbes and can act as antimicrobial factors that modulate microbial interaction with corneal epithelial cells (Taube et al., 2015). Kugadas et al. showed that the presence of the OSM strengthens the innate barrier by markedly raising tear levels of immune effectors such as secretory IgA and complement proteins (Kugadas et al., 2016). Germ free mice developed more severe *Pseudomonas aeruginosa* keratitis than conventionally housed controls, revealing a link among gut microbes, the OSM, and ocular defense. Colonizing the conjunctival sac of infected germ-free animals with a single isolate of CNS recovered from human conjunctival swabs restored resistance to infection, underscoring a potential role of the OSM in tuning the local microenvironment and preserving ocular health. Intestinal commensals also contribute to maintaining baseline tear SIgA concentrations. Kugadas et al. observed that antibiotic-driven depletion of gut commensals simultaneously lowers tear SIgA protein and IgA transcript levels at the ocular surface (Kugadas et al., 2017). This finding prompted them to propose a gut-eye immune axis that links intestinal microbes to ocular immune status. Subsequent work has corroborated the concept: the gut microbiota has been reported to influence ocular health by modulating systemic immunity, preserving the blood-retinal barrier and delivering beneficial microbial metabolites. Conversely, dysbiosis has been associated with ocular inflammation, tissue injury and disease progression (Tîrziu et al., 2024).

### 4.2 Immune tolerance

The OSM has been proposed to safeguard ocular tissue by promoting immunological tolerance, thereby averting collateral damage from excessive immune reactions, although direct evidence for this mechanism remains limited (Kate and Basu, 2022). Among CD4^+^ T lymphocytes, T helper 17 cells (Th17) and regulatory T cells exert opposing regulatory influences. When naive CD4^+^ T cells are exposed to TGF-β together with IL-6 or IL-21, the orphan nuclear receptor RORγt is upregulated, driving differentiation into the Th17 lineage. These Th17 cells are pro- inflammatory helper T cells that characteristically secrete IL-17. Regulatory T cells (Treg) are specialized immunoregulatory lymphocytes that normally exert anti-inflammatory effects. They secrete IL-10 and TGF-β, counterbalance pro-inflammatory mediators, suppress Th17 responses and thereby help maintain immunological tolerance. The ratio between these two CD4+ subsets is critical for immune homeostasis. Its disruption is linked to numerous inflammatory and autoimmune disorders (Wagner et al., 2021). It is therefore plausible that Treg cells play a pivotal role in the tolerance induced by the OSM. Through reciprocal interaction with Treg cells, the microbiota may safeguard ocular immune equilibrium. At present, however, detailed insight into how distinct T-cell subsets shape, and are shaped by, the OSM remains scarce and awaits further investigation.

### 4.3 Potential pathogenicity of OSM

Under physiological conditions the OSM remains in a balanced state that rarely precipitates infection or inflammation. When the local milieu is disturbed, however, these same organisms can assume a pathogenic role. A clear example is postoperative invasion through a surgical incision, which may lead to infectious endophthalmitis. Following cataract surgery the most frequently isolated causative agents are CNS, *Staphylococcus aureus* and streptococci (Aragona et al., 2021). Distinct pathogens elicit divergent therapeutic responses. Endophthalmitis by CNS generally carries a favorable visual prognosis, whereas infections caused by streptococci, *Staphylococcus aureus* or Gram-negative bacilli are associated with poorer visual outcomes (Durand, 2017). CNS adhere to intraocular structures and form biofilms that impair antibiotic penetration. Moreover, resistance plasmids can spread among these Staphylococci through transformation or transduction, disseminating antimicrobial resistance (Argemi et al., 2019). Recent work has revealed that Streptococci evade host surveillance through multiple strategies. Surface adhesins such as M protein and fibronectin binding protein mediate tight association with host cells, facilitating immune escape and promoting infection. *Streptococci* secrete an array of exotoxins and enzymes such as streptolysins and hyaluronidase. These factors blunt host immune responses and facilitate bacterial dissemination. In addition, Streptococci modulate the host inflammatory cascade to promote their own survival and replication (Lannes-Costa et al., 2021; Cunningham, 2000). Pathogenicity of *Staphylococcus aureus* is linked to numerous virulence determinants. Its cell wall contains peptidoglycan and protein A, which help the organism resist host immunity. The species also produces toxins including α-hemolysin, β-hemolysin and staphylococcal enterotoxins. These toxins damage host cells, leading to tissue injury and pronounced inflammation. The organism also releases a panel of enzymes, among them coagulase and hyaluronidase. These enzymes degrade extracellular matrices, promote bacterial spread and facilitate abscess formation. Moreover, *Staphylococcus aureus* can construct a biofilm that enhances both antibiotic tolerance and resistance to host immune attack (Ahmad-Mansour et al., 2021; Cheung et al., 2021). In a cross-sectional survey of 347 patients with ocular surface infection, Fenta et al. (2022) found that Gram-positive pathogens accounted for 34.3% of all cases, with *Staphylococcus aureus*, CNS and *Streptococcus pneumoniae* being the most frequently isolated organisms. It is noteworthy that the same species are frequently detected in the OSM of healthy individuals. However, the vast majority of isolates reported in the above study were multidrug-resistant. Moreover, the investigation identified the prophylactic or indiscriminate use of topical antibiotics as a significant risk factor for ocular surface infection. These observations suggest that antibiotic overuse may disrupt the native microbial equilibrium, potentially allowing resistant strains to expand and assume a pathogenic phenotype. When host immunity declines or the epithelial barrier is compromised, these organisms have the potential to penetrate ocular tissues and have been implicated in clinically overt infection (Taube et al., 2015). Viruses can also shape ocular surface health and immunity. Clinical studies show that viral infection can alter the composition of the OSM and modulate local immune responses (Mahjoub et al., 2022). When a virus invades, the ocular immune system is rapidly activated. An excessive reaction may damage epithelial tissue and amplify inflammation, creating a vicious cycle (Smith et al., 2022). Recognizing the potential role of the OSM and preserving its homeostasis may therefore be important for maintaining a healthy ocular surface.

## 5 Limitations of current OSM research

These advances notwithstanding, the field remains constrained by limitations that complicate both interpretation and cross-study comparison. The ocular surface is an ultra-low-biomass niche: a conjunctival swab yields orders of magnitude fewer microbial cells than gut or oral samples, leaving sequence data vulnerable to contaminating DNA from sampling devices, extraction kits and laboratory reagents (Doan et al., 2016; Delbeke et al., 2023). Without negative controls, blank swabs and reagent blanks processed in parallel, genuine community members cannot be reliably distinguished from contamination. Compounding this problem, the field lacks standardized protocols. Studies vary in sampling site and device, sampling depth, DNA extraction and purification, the 16S rRNA variable regions targeted, sequencing platforms, and bioinformatic pipelines and reference databases, each a source of systematic bias. Head-to-head comparison has shown that the extraction protocol alone can produce significantly different community profiles, even after computational decontamination (Delbeke et al., 2023).

Beyond this, reproducibility is limited. For instance, many taxa show substantial differences in relative abundance across cohorts. In some cases, the detectability of these taxa also varies greatly between study groups. This variability reflects genuine biological influences (geography, age, sex, lifestyle and health status) as much as the methodological heterogeneity noted above (Huang et al., 2016; Deng et al., 2020). Cross-sectional profiling poses a further constraint: it cannot separate resident members of the OSM from transient microorganisms deposited by the environment, skin or contact lenses. Defining a bona fide resident community will require longitudinal sampling coupled with absolute quantification (Ozkan et al., 2017). Many studies largely rely on animal models, small observational cohorts and indirect systemic correlations. Therefore, associations between OSM and ocular surface diseases, including dry eye disease, meibomian gland dysfunction and infectious keratitis, must be interpreted as correlative rather than causal. Establishing causality will require standardized longitudinal cohorts, animal models with sterile and colonized microbiota, and interventional studies targeting microbial modulation.

## 6 Conclusion

As an integral component of the ocular microecological system, the OSM has attracted intensifying research interest in recent years. This review systematically summarizes current knowledge on the compositional profile and diversity of OSM and delineates its distribution patterns in healthy populations. We further dissect how environmental, age-related, sex-specific and health-associated variables shape microbial community structure. The manuscript then analyzes the intricate interplay between OSM and the host immune system, emphasizing its proposed roles in preserving ocular immune homeostasis, promoting immune maturation and orchestrating immunological tolerance. Under specific circumstances, however, members of the OSM may assume a pathogenic role and have been implicated in the initiation and progression of ocular surface infections and inflammatory disorders. With the development of multi-omics technologies, future research on OSM will be more in-depth, and its application prospects in early diagnosis, personalized treatment, and OSM intervention of ocular surface diseases are broad. It should nevertheless be stressed that most of the current evidence remains associative, and causal relationships between the OSM, ocular immunity and disease await confirmation in standardized, longitudinal and interventional studies. Collectively, the OSM may represent not only a sentinel indicator of ocular surface health, but also a valuable entry point for elucidating disease mechanisms and developing novel therapeutic approaches.
